# Lymph node metastasis prediction and biological pathway associations underlying DCE-MRI deep learning radiomics in invasive breast cancer

**DOI:** 10.1186/s12880-024-01255-y

**Published:** 2024-04-16

**Authors:** Wenci Liu, Wubiao Chen, Jun Xia, Zhendong Lu, Youwen Fu, Yuange Li, Zhi Tan

**Affiliations:** Radiology Imaging Center, The Affiliated Hospital of Guangdong Medical University, 524001 Zhanjiang, Guangdong Province P. R. China

**Keywords:** Invasive breast cancer, Lymph node metastasis, Radiomics, Deep learning, Biologic pathway

## Abstract

**Background:**

The relationship between the biological pathways related to deep learning radiomics (DLR) and lymph node metastasis (LNM) of breast cancer is still poorly understood. This study explored the value of DLR based on dynamic contrast-enhanced magnetic resonance imaging (DCE-MRI) in LNM of invasive breast cancer. It also analyzed the biological significance of DLR phenotype based on genomics.

**Methods:**

Two cohorts from the Cancer Imaging Archive project were used, one as the training cohort (TCGA-Breast, *n* = 88) and one as the validation cohort (Breast-MRI-NACT Pilot, *n* = 57). Radiomics and deep learning features were extracted from preoperative DCE-MRI. After dual selection by principal components analysis (PCA) and relief methods, radiomics and deep learning models for predicting LNM were constructed by the random forest (RF) method. A post-fusion strategy was used to construct the DLR nomograms (DLRNs) for predicting LNM. The performance of the models was evaluated using the receiver operating characteristic (ROC) curve and Delong test. In the training cohort, transcriptome data were downloaded from the UCSC Xena online database, and biological pathways related to the DLR phenotypes were identified. Finally, hub genes were identified to obtain DLR gene expression (RadDeepGene) scores.

**Results:**

DLRNs were based on area under curve (AUC) evaluation (training cohort, AUC = 0.98; validation cohort, AUC = 0.87), which were higher than single radiomics models or GoogLeNet models. The Delong test (radiomics model, *P* = 0.04; GoogLeNet model, *P* = 0.01) also validated the above results in the training cohorts, but they were not statistically significant in the validation cohort. The GoogLeNet phenotypes were related to multiple classical tumor signaling pathways, characterizing the biological significance of immune response, signal transduction, and cell death. In all, 20 genes related to GoogLeNet phenotypes were identified, and the RadDeepGene score represented a high risk of LNM (odd ratio = 164.00, *P* < 0.001).

**Conclusions:**

DLRNs combining radiomics and deep learning features of DCE-MRI images improved the preoperative prediction of LNM in breast cancer, and the potential biological characteristics of DLRN were identified through genomics.

## Background

Medical imaging such as computer tomography, magnetic resonance imaging (MRI), and ultrasound are important and non-invasive examination tools used in cancer diagnosis and treatment. Medical images carry tumor information for clinical decision-making in two forms: “semantic” and “agnostic” phenotypes [1, 2]. Semantic features are mainly based on the extent of visualization by the naked eye; they have low information utilization but are highly explanatory. Agnostic phenotypes are mainly based on computer visualization of tumor information in mathematical form; they are informative and highly utilized, but their interpretability is debatable.

The application of semantic phenotypes in tumor treatment process has been clarified. For example, an adjacent vessel sign was associated with axillary lymph node metastasis (LNM), increased Ki-67 index, and lymphovascular infiltration, representing poorer prognosis for patients [3]. In contrast to semantic phenotypes, agnostic phenotypes not only quantify morphological phenotypes, but also break through the limitations of the naked eye to quantify information at deeper level-even that related to the genetic level. For example, based on 91 preoperative breast cancer MRI images, quantitative radiomics was found to replicate the representation of tumor size and BI-RADS imaging phenotypes visually extracted by radiologists [4]; MRI-based quantification represented phenotypes of size and shape appropriately correlated with proliferative and apoptotic pathways [5].

Agnostic phenotypes have two main forms of quantification: radiomics and deep learning [6, 7]. Different techniques capture their own specific information, and the fusion of information between techniques can further improve the knowledge of the target. Multitype studies based on radiomics and/or deep learning features have been conducted to evaluate LNM in breast cancer. For example, in one multi-center study, a radiogenomics model combining radiomics features and genomics features improved the performance of predicting LNM in breast cancer [8]; in another based on 110 MRI images from a single center, potential pathways for the genetic mechanism of deep learning imaging phenotypes were identified [9]. The biological significance of the radiomics feature phenotype had been analyzed in multiple studies, but deep learning feature phenotypes were only identified during the image recognition process, and the quantification of specific features was not mentioned. In contrast to radiomics phenotypes, deep learning phenotypes are more inclined to be quantified by automatic learning, and the biological significance of their representation still needs to be further explored.

This study was focused on the development of deep learning radiomics nomograms (DLRNs) based on two MRI image cohorts to explore DLRNs association with LNM in invasive breast cancer. The biological significance represented by deep learning radiomics (DLR) phenotype was also explored based on genomics.

## Methods

### Image cohorts

Two MRI image cohorts of invasive breast cancer were downloaded from the Cancer Imaging Archive (TCIA) project [10]. One was the training cohort (The Cancer Genome Atlas-Breast, TCGA, *N* = 108), and the other was the validation cohort (Breast-MRI-NACT Pilot, *N* = 64). The inclusion criteria for the image cohorts were preoperative dynamic contrast enhanced magnetic resonance imaging (DCE-MRI), which only used 1.5 Tesla magnet strength using GE scanners and protocols (GE Medical Systems, Milwaukee, WI, USA) for clarifying the potential difference between scanner and image protocol (training cohort, *n* = 93; validation cohort, *n* = 64). In order to explore the biological characteristics corresponding to the imaging phenotype, the transcriptome data of the training cohort were downloaded from TCGA project (UCSC Xena online database, FPKM format) matched by TCIA, and the biological characteristics represented by the imaging phenotype were analyzed based on messenger RNA (mRNA) molecular correlation [11]. Cases that could not be located or were ambiguous (*n* = 4) or were without transcriptome data (*n* = 1) were also excluded from the training cohort. Cases that could not be located or were ambiguous (*n* = 7) were also excluded from the validation cohort. Finally, under the condition that two radiologists checked them without ambiguity, 145 patients with breast cancer were included in the image cohorts (training cohort, *n* = 88; validation cohort, *n* = 57). The mean age was 51.16 ± 11.01 years, with an age range of 29–82 years.

### Image segmentation and phenotype quantification

Breast DCE-MRI images were collected using dual breast coils on 1.5-T scanners. The imaging protocol was based on T1-weighted gradient echo sequences of gadolinium-based contrast agents [10]. The sagittal T1WI DCE-MRI images of the cohorts were imported into ITK-snap (version 3.68) software, with the same window width (800) and window level (1600) set for each. To improve discrimination between phenotypes, the acquisition areas were resampled to specific isotropic resolutions (1 mm, 1 mm, 1 mm) [12]. Two radiologists (5 and 10 years of experience) identified and ruled the boundaries of the tumor lesions, without earlier information on the pathology and without clinical information. In the Python environment, 1781 phenotypes including size, shape, edge, and internal structure information were extracted from the regions of interest (ROIs) by PyRadiomics extractor [13].

For deep learning phenotypes, in the case of the above ITK-snap software segmentation image, based on the maximum diameter and clear level of the lesions, two kinds of deep learning networks (GoogLeNetNet and ResNet50) were used for feature extraction in the Python environment [14].

### Development and validation of deep learning radiomics predictive models

To increase the comparability of phenotypes, Z-score method was applied to normalize all radiomics or deep learning phenotypes. Both radiomics and deep learning phenotypes were in thousands, in high dimensions, with mixed information and redundancy, so it need to identify valuable phenotypes closely related to LNM for reducing the possibility of overfitting. For this reason, principal components analysis (PCA) and relief algorithm were applied for dimensionality reduction, and random forest (RF) was applied for modeling [15–17]. First, in the training cohort, PCA primarily recombined a new set of uncorrelated composite variables from all phenotypes and extracted a few of the composite variables that reflected the original state of the image phenotypes as faithfully as possible. Then, the extracted phenotypes were provided to the relief algorithm to select an optimal modeling subset through the LN state, and present it to the RF machine learning algorithm to construct predictive models. The optimal models were selected after 5-fold cross validation, denoted as radiomics or deep learning signature. Repeating this process, radiomics models, GoogLeNet models and ResNet50 models were constructed, respectively. To this end, radiomics and deep learning phenotypes were integrated to construct the DLRNs [18].

### Identification of biological significance of deep learning radiomics

**Fig. 1:**
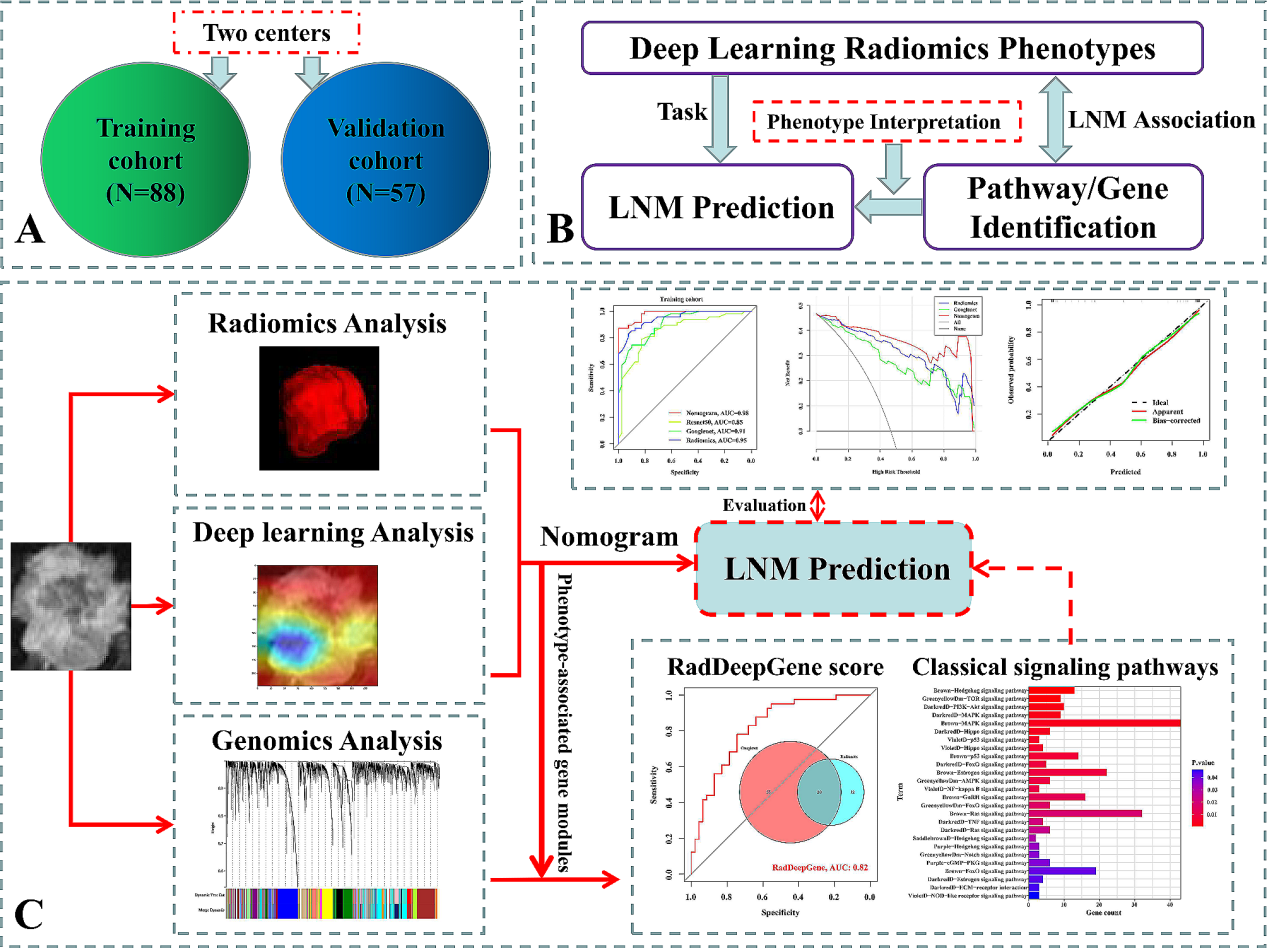
Overview of the study. (**A**) Cohorts downloaded from TCIA. (**B**) Overall design of this study. (**C**) Flow chart of this study

In the training cohort, for the mRNA matrix, log2(x + 1) was used to normalize all genes and remove genes with mean values less than 1 from all samples, leaving 11,687 genes. Weighted gene co-expression network analysis (WGCNA) is a system biology approach to describing gene association patterns. It can identify highly synergistic variants in genomics modules and is based on the association of genomics modules with phenotypes [19]. To better understand the biological significance represented by tens of thousands of genes, WGCNA analysis of the 11,687 genes was implemented based on R language. Module eigengenes (MEs) were the expression profiles of module genes; the minimum number of genes per module was set to 60, the sensitivity was 3, and the module merging threshold was 0.25 [20]. In addition, the radiomics signature and GoogLeNet signature were classified into high and low risk groups with median values, and their correlation with different modules was analyzed. Further, the biological significance of the associated modules was analyzed using the Kyoto Gene and Genome Encyclopedia (KEGG) database [21]. Gene set variation analysis (GSVA) calculated genes of the same significance or function grouped into a single enrichment score in order to assess changes in the activity of the pathway/function in which the genes were located [22]. GSVA was used to study 36 genetic pathways in the KEGG database, and their association with the imaging phenotype was analyzed based on Pearson correlation coefficient (PCC). The technical flowchart of the study is presented in Fig. 1.

### Statistic analysis

SPSS (version 17.0), (version 4.20), and MedCalc software were used for statistical analysis and plotting. Measurement data were compared between groups by using the Mann-Whitney U test. The counting data were compared between groups using the chi-square test. The performances of the models were evaluated using the receiver operating characteristic curve (ROC) cure and the area under the curve (AUC), accuracy (ACC), sensitivity (SEN), and specificity (SPEC) were calculated. Based on MedCalc software, the Delong test was used to analyze the merits of the different ROC curves [23]. In addition, the net reclassification index (NRI) and the integrated discrimination index (IDI) were calculated based on the “PredictABEL” package to compare the discrimination performance of the different prediction models [24, 25]. The Hosmer-Lemeshow test was used to analyze the consistency of the DLRNs. Decision curve was used to analyze the clinical utility of the DLRN. *P* < 0.05 was considered statistically significant.

## Results

### Lymph node status in image cohorts

**Fig. 2:**
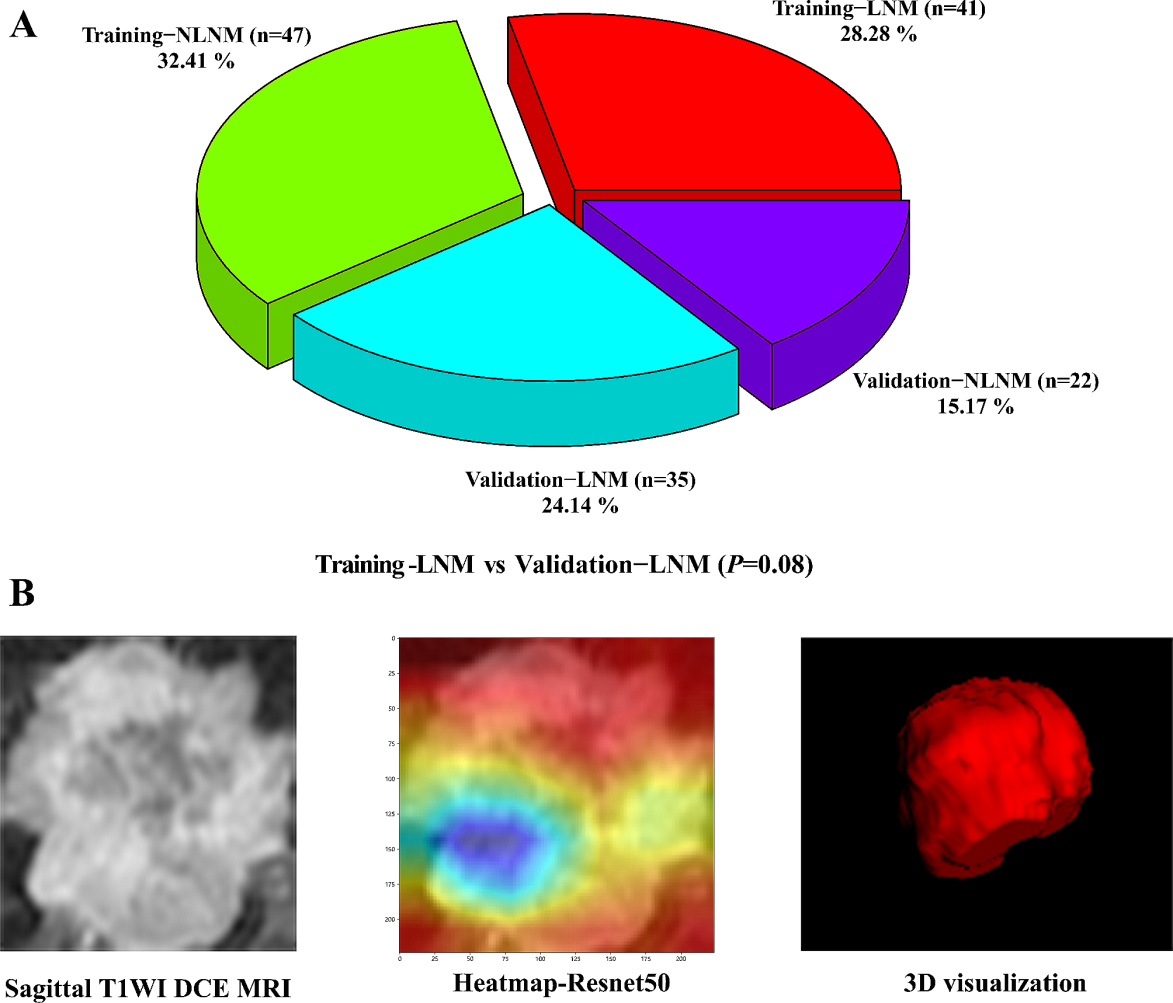
Lymph node status in image cohorts. (**A**) 145 LN distribution of invasive breast cancer. There was no statistically significant difference between cohorts (chi-square test, *P* = 0.08). (**B**) Typical LNM example. Deep learning phenotypes were based on 2D image extraction, radiomics phenotypes were based on 3D image extraction

The training cohort included 88 invasive breast cancer patients with a 46.59% LNM positivity rate; and the validation cohort included 57 invasive breast cancer patients with a 61.40% LNM positivity rate, which was not statistically significant (chi-square test, *P* = 0.08) (Fig. 2).

### Development and validation of deep learning radiomics predictive models

In the training cohort, after 1781 phenotypes were selected by the dual method of PCA and relief, 41 phenotypes were submitted to the RF machine learning algorithm to develop the radiomics model. The radiomics model yielded ACC, SEN, SPEC, and AUC of 0.88, 0.93, 0.83, and 0.95 (95% confidence interval (CI), 0.91–0.98), respectively, in the training cohort; and 0.79, 0.77, 0.82, and 0.83 (95% CI, 0.69–0.94), respectively, in the validation cohort.

From 1024 GoogLeNetnet and 2048 ResNet50 deep learning phenotypes, 67 and 39 phenotypes were selected in the same way to construct prediction models. The GoogLeNet model yielded ACC, SEN, SPEC, and AUC of 0.82, 0.90, 0.74, and 0.91 (95%CI, 0.84–0.96), respectively, in the training cohort; and 0.77, 0.86, 0.64, and 0.77 (95% CI, 0.64–0.90), respectively, in the validation cohort. The ResNet50 model yielded ACC, SEN, SPEC, and AUC of 0.81, 0.83, 0.79, and 0.85 (95%CI, 0.76–0.94), respectively, in the training cohort; and 0.70, 0.66, 0.77, and 0.71 (95% CI, 0.56–0.86), respectively, in the validation cohort.

In the training cohort, in the prediction reliability of the different models were explored by Delong test. The radiomics model and the GoogLeNet model (*P* = 0.25), the ResNet50 model (*P* = 0.05) had no statistically different. In addition, there was no statistical difference between the GoogLeNet model and the ResNet50 model in the comparison of the two deep learning models (*P* = 0.30). The results of the validation cohort were consistent with the training cohort. However, in terms of sensitivity, the GoogLeNet model was more capable of predicting LNM than the ResNet50 model in the training cohort and validation cohorts. Therefore, GoogLeNet model was selected for further analysis.

**Fig. 3:**
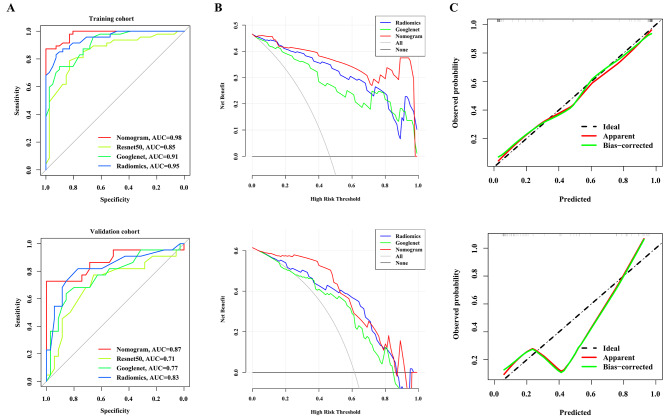
The performance of deep learning radiomics models. (**A**) ROC curve analysis. (**B**) DCA analysis. (**C**) Calibration curve analysis

Development of the DLRN proceeded by integrating radiomics model and GoogLeNet model through a logistic regression algorithm. The DLRN yielded ACC, and AUC of 0.93 and 0.98 (95%CI, 0.93-1.00), respectively, in the training cohort; and 0.90 and 0.87 (95% CI, 0.76–0.95), respectively, in the validation cohort (Fig. 3A; Table 1). The Delong test showed that the DLRN was statistically different from the radiomics model (*P* = 0.04) and the GoogLeNet model (*P* = 0.01) in prediction effect, indicating that the image information through multiple methods has some complementary effect to improve the prediction effect. After the calculation of NRI and IDI, it was verified that the DLRN improved the prediction effect compared with the single radiomics model (training cohort, NRI > 0, *P* = 0.68, IDI: 12.00%, *P* = 0.004; validation cohort, NRI > 0, *P* < 0.01, IDI: 14.00%, *P* < 0.01). DCA proved that the net income of the DLRN was higher than that of the radiomics or GoogLeNet model, indicating that the treatment strategies predicted based on the DLRN had better clinical effectiveness (Fig. 3B). The Hosmer-Lemeshow test indicated good agreement between DLRN predictions and actual LNM (training cohort, *P* = 0.51; validation cohort, *P* = 0.19) (Fig. 3C).

**Table 1:** Performance of diferent models for predicting LNM in the training and validation cohorts

Model	Training cohort					Validation cohort			
	Radiomics	GoogLeNet	ResNet50	DLRN		Radiomics	GoogLeNet	ResNet50	DLRN
AUC	0.95	0.91	0.85	0.98		0.83	0.77	0.71	0.87
95%CI	0.91–0.98	0.84–0.96	0.76–0.94	0.93-1.00		0.69–0.94	0.64–0.90	0.56–0.86	0.76–0.95
ACC	0.88	0.82	0.81	0.93		0.79	0.77	0.70	0.90
SEN	0.93	0.90	0.83	1.00		0.77	0.86	0.66	1.00
SPEC	0.83	0.74	0.79	0.73		0.82	0.64	0.77	0.85
PPV	0.83	0.76	0.77	0.87		0.87	0.79	0.82	0.85
NPV	0.93	0.90	0.84	1.00		0.69	0.74	0.59	1.00

**Fig. 4:**
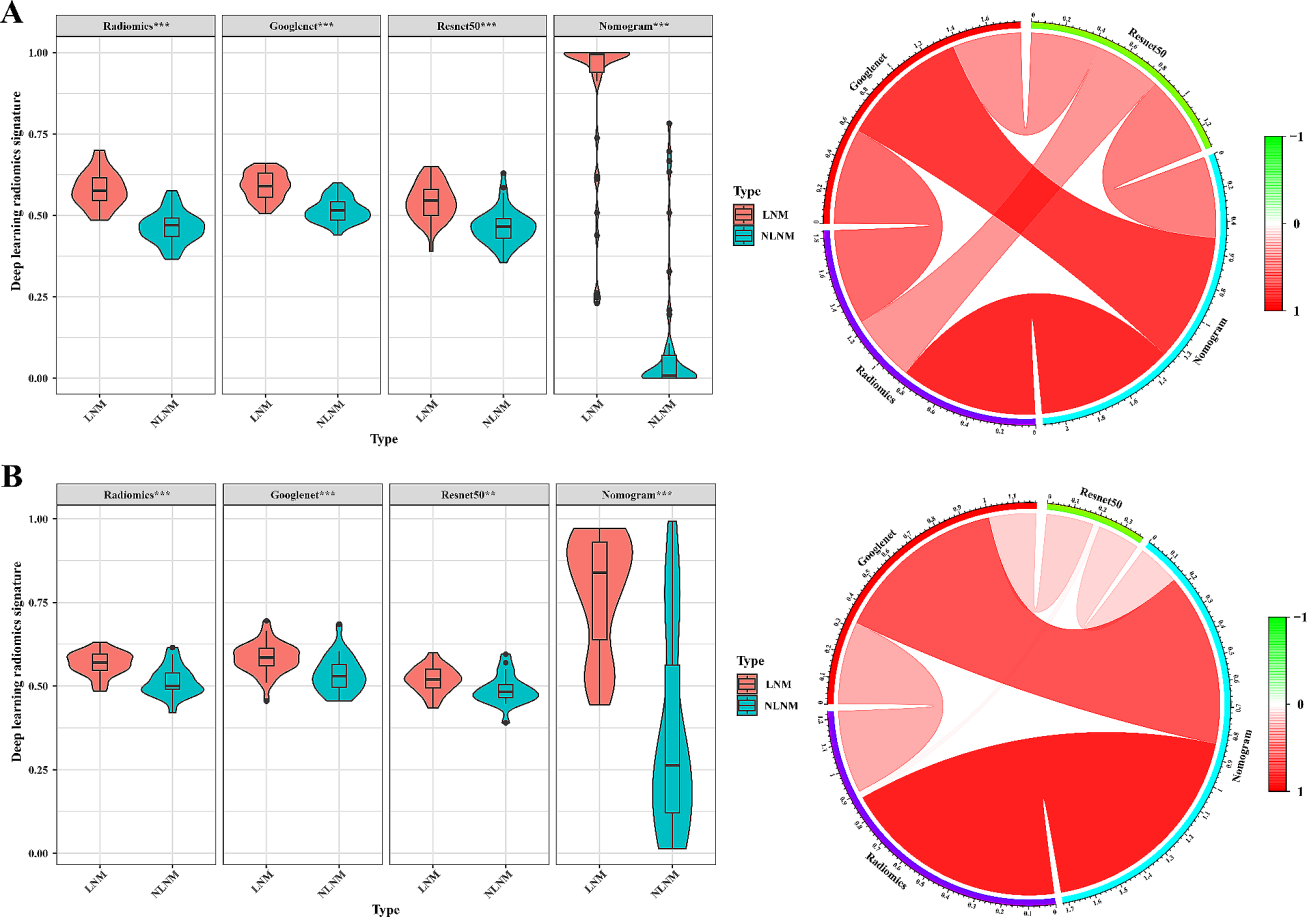
Association of deep learning radiomics signatures. (**A**) Training cohort. (**B**) Validation cohort. Distribution difference of signatures in LN status of each cohort (Mann-Whitney U test, * *P*<0.05, ** *P*<0.01, ***P<0.001). Model signatures were correlated in the training cohort, especially in the radiomics and GoogLeNetnet signatures (PCC, *r* = 0.59, *P* < 0.01), and also in the validation cohort (PCC, *r* = 0.32, *P* = 0.01)

The LN status distributions of model signatures in all cohorts were significantly different (Mann-Whitney U test, *P* < 0.05). In addition, all signatures were correlated in the training cohort, especially for the radiomics and GoogLeNet signatures (PCC, *r* = 0.59, *P* < 0.01), and also in the validation cohort (PCC, *r* = 0.32, *P* = 0.01) (Fig. 4).

### Identification of Biological significance of deep learning radiomics

**Fig. 5:**
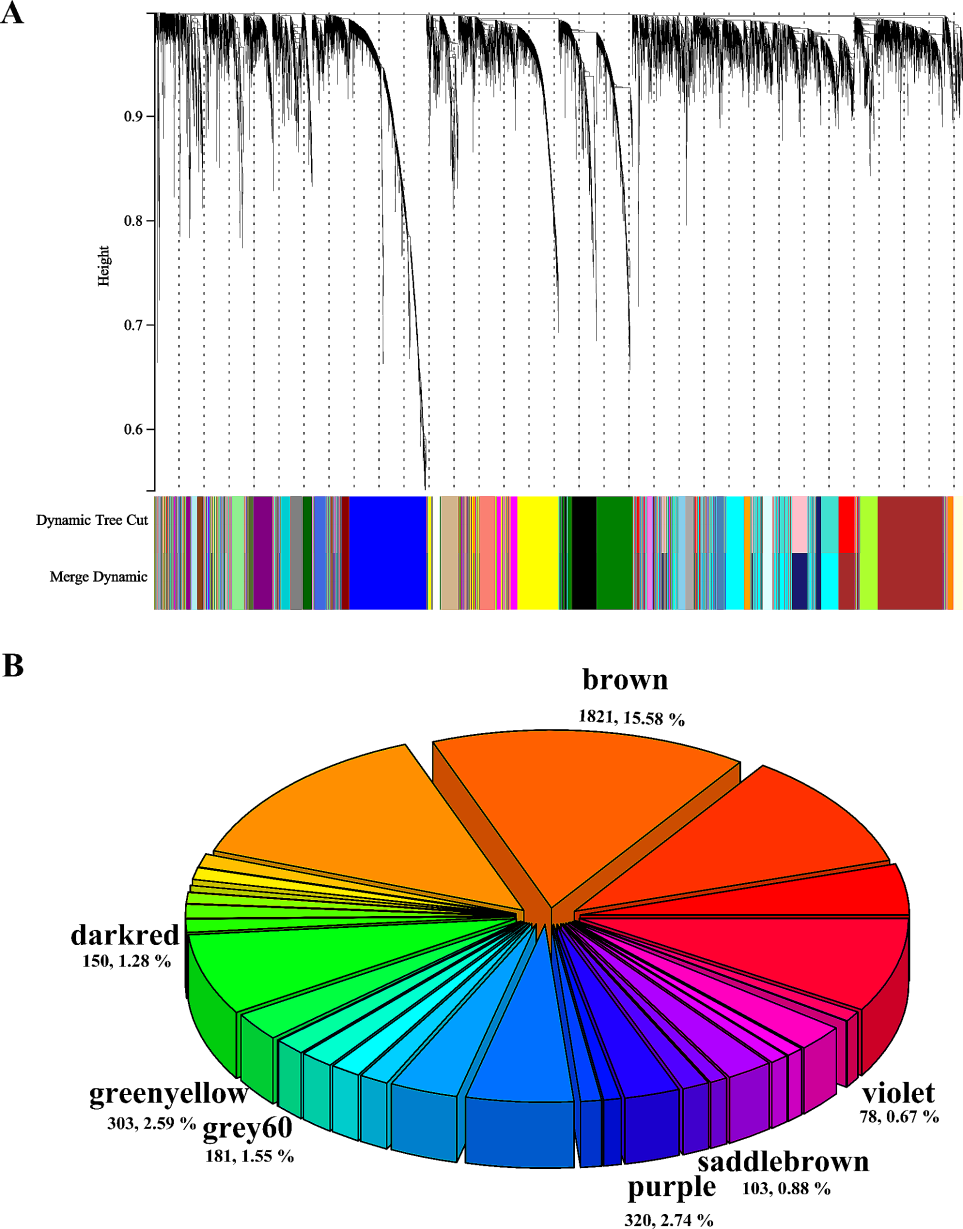
Gene coexpression module construction. Through WGCNA analysis (**A**), a total of 32 gene modules were constructed (**B**)

**Fig. 6:**
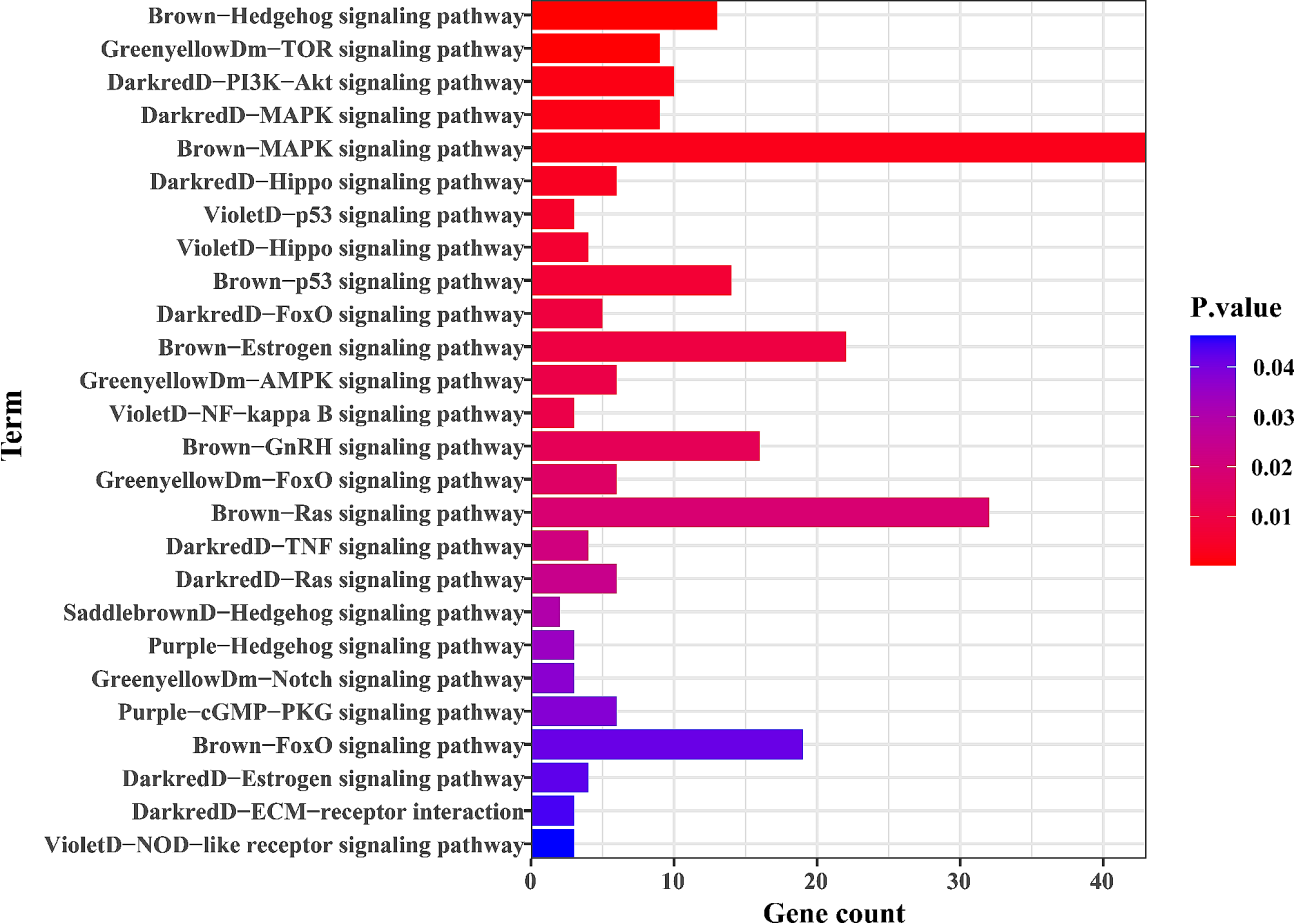
Identification of biological significance of deep learning radiomics

In the training cohort, 32 gene modules were constructed by WGCNA analysis, and the results were shown in the Fig. 5A and B. Two gene modules (brown module; purple module) were associated with the radiomics signature high-risk group (Pearson correlation *r* = 0.22, *P* = 0.04 for the brown module; Pearson correlation *r* = 0.23, *P* = 0.03 for the purple module). Surprisingly, six gene modules were associated with the GoogLeNet signature high-risk group, with the green-yellow module having the strongest positive correlation (Pearson correlation *r* = 0.31, *P* < 0.01) and the dark-red module having the strongest negative correlation (Pearson correlation *r*=-0.29, *P* < 0.01). Interestingly, the brown module was associated with both the radiomics and GoogLeNet signature. The core genes associated with the high-risk group were identified from the related gene module, resulting in 32 genes identified from the radiomics-related module and 75 genes identified from the GoogLeNet-related module, intersecting 20 genes to construct a DLR-related gene (RadDeepGene) score that also reliably predicted LNM (AUC = 0.82, SEN, 0.78, SPEC, 0.74; risk coefficient, OR = 164.00, *P* < 0.001). The biological significance of signature representation was further explored by KEGG. Different pathways were enriched in radiomics-related and GoogLeNet-related gene modules, mainly focusing on classical signaling pathways, and the results were shown in Fig. 6. The results for the brown module deserved more attention. It was closely related to Hedgehog, MAPK, P53, Ras, and FoxO signaling pathways. In addition, estrogen and GnRH signaling pathway indicated that hormone secretion related pathways were also closely related to the signatures. Hedgehog and FoxO signaling pathways were related to signatures in multiple modules.

To further explore the biological significance of DLR phenotypes with breast cancer invasion and metastasis, genetic and molecular functions associated with tumor cell growth and death, immune response, cell communication and signal transduction pathways were identified by GSVA. Unfortunately, in that the radiomics signature was not associated with these genetic and molecular functions; instead, GoogLeNet signature was associated with cell death (*r*=-0.27), immune response (r, -0.28-0.23), signal transduction (*r*=-0.21), and especially immune response, with the presence of five signaling pathways. Excitingly, MAPK signaling pathway and NOD-like receptor signaling pathway showed correlations with the GoogLeNet signature in both KEGG enrichment analysis and association analysis.

## Discussion

DLR MRI agnostic phenotypes have been shown to be potential alternative markers for predicting breast cancer LNM, but the biological significance of DLR phenotypes, especially deep learning phenotypes, remains poorly understood. In this study, first, the potential value of radiomics and deep learning phenotypes to predict breast cancer LNM was validated based on two cohorts, both with AUCs above 0.70 and accuracy above 0.70 in the validation cohort. Undoubtedly, the DLRN developed based on radiomics-deep learning phenotypes improved prediction after the Delong test and NRI calculation as in other similar studies. Then, potential biological pathways associated with LNM were revealed, with a special focus on typical signaling pathways as well as pathways for cell growth and death, immune response, cell communication, and signal transduction. Surprisingly, both radiomics and deep learning phenotypes were associated with brown gene modules at high risk of LNM, with classical pathways of signal transduction, such as the MAPK signaling pathway and with signaling pathways of immune response, such as the NOD-like receptor signaling pathway. In addition, 20 key genes associated with DLR phenotypes were identified, and the constructed RadDeepGene score showed the same medium to high predictive effect (AUC = 0.82, OR = 164.00, *P* < 0.001). The findings indicated that DLRN combining radiomics and deep learning features of DCE-MRI images improved the performance of preoperative prediction of LNM in breast cancer, and the potential biological characteristics of DLRN were identified through genomics.

This study could be said to summarize the characteristics of previousstudies and to further expand their results. Even though DLR studies had combined deep learning and radiomics features, but there were still lack of studies on DLR biological characteristic analysis, which was the novelty of this study. First, multiple studies had analyzed the predictive value of deep learning and radiomics phenotypes, but ignored the interactions between the two and the variability of phenotypes quantified by different deep learning algorithms. This study both verified that radiomics and deep learning phenotypes offer complementary information and found an information overlap that increases the interpretability of deep learning phenotypes. In addition, most previous studies had used the VGG16 deep learning algorithm to extract image information [26]. In contrast, this study used the GoogLeNet and ResNet50 deep learning algorithms and compared the differences in the information extracted by them. No difference was observed in the effectiveness of the models developed by these two deep learning phenotypes. Second, this study revealed the biological significance of deep learning radiomic phenotypes transferred with LNM based on radiogenomics. In contrast to similar studies, the biological significance of deep learning phenotypes was still poorly understood. Moreover, most radiogenomics studies had analyzed the biological significance represented by a large number of differentially expressed genes, but ignored the interactions between genes. This study mapped imaging phenotypes to gene modules based on WGCNA, which may enhance its reproducibility and interpretability. In addition, most previous studies had focused on relatively broad imaging-related pathways, whereas specific pathways associated with deep learning of radiogenomic phenotypes, especially immune-related pathways, were dissected in the present study.

In a past investigation, it was found that DLR was mainly used in the three areas of breast tumor diagnosis and identification, LNM, and neoadjuvant chemotherapy efficacy; for example, based on the handmade radiomics phenotypes and deep phenotypes extracted from 3062 DCE-MRI images for predicting LNM of breast cancer, the support vector machine model achieved a moderate prediction effect [27]. Based on radiomic phenotypes and deep learning phenotypes from DCE-MRI images before and after neoadjuvant chemotherapy to predict pathological complete remission, the AUC reached 0.9 [28]. In this study, radiomics phenotypes seem to be more effective than deep learning phenotypes, similarly to previous studies. For example, based on the RF machine learning algorithm, the radiomics model exhibited better discrimination than the deep learning model (AUC, 0.86 vs. 0.79; accuracy, 0.78 vs. 0.73) [29]. The DLRN developed based on DLR phenotypes showed prediction results similar to those in previous studies. For the degree of information quantification of the images, this study quantified 1781 radiomics phenotypes, which was a larger amount compared to some previous studies. However, the thousands of deep learning phenotypes quantified based on GoogLeNet and ResNet50 were less informative compared to vgg16. In addition, for model evaluation, the Delong test and NRI/IDI calculations were used to double-validate the differences in the reliability of the different models, and NRI was found to suggest differences between models in the case of a negative Delong test. For example, the Delong test found no significant difference between the radiomics model and the GoogLeNet model, but the NRI found that the radiomics model improved the effect over the GoogLeNet model (*P* = 0.008).

The relationship between image phenotypes and biological pathways has been shown in numerous studies, suggesting the possibility of observing cancer-related pathways in a non-invasive manner by DCE-MRI. For example, prognostic radiomic phenotypes of glioblastoma cells could be classified into four types based on the potential pathways of radiomic phenotypes, reflecting key biological processes related to immune regulation, tumor proliferation, therapeutic response and cellular functions, all of which affect patient survival outcomes [30]; genes in the cell cycle pathway exhibited significant associations with MRI imaging phenotypes [31]. The inhomogeneous enhancement phenotypes of tumor-adjacent parenchyma in MR imaging were associated with tumor necrosis signaling pathways [32]. However, there were still limited studies on the biological characteristics of imaging features related to LNM. For example, LNM was evaluated based on the deep learning features extracted from MRI images of 110 breast cancer patients, and the potential biological characteristics of deep learning features were analyzed, among which fatty acid metabolism, insulin signaling pathway, phynylalanine metabolism, RNA degradation, and tyrosine metabolism were the first five related pathways [9]. WGCNA has proven to be a useful tool for identifying gene modules in radiogenomic analysis of breast tumors, where MRI phenotypes were associated with seven gene modules, including two radiomics phenotypes and six GoogLeNet phenotypes in this study. Referring to previous studies, DLR phenotypes of tumor progression closely signaling pathways were analyzed [5], such as cell growth and death, immune response, cell communication, and signal transduction, and the results showed that image phenotypes were most closely related to immune response. This study was not consistent with the results of previous studies, mainly due to different analytical methods. Unlike previous studies on KEGG analysis of differentially related differential genes. This study mainly conducted a precise association analysis based on pathways closely related to tumors that had been organized.

In this study, radiomics also had inherent limitations. First, the study was a small retrospective study that lacked the generalization effect of external cohort enhancement results despite the use of two cohorts from different sources. Second, although both cohorts used the 1.5T GE Medical System, there were potential differences in image protocols between the two. Third, the clinical characteristics of the two cohorts were not identical and there may be uncertainty about other treatments prior to pathologic determination of LNM. Fourth, although cross-scale associations were identified between radiomics and genomics, their causality was not been validated in molecular or clinical trials. Finally, even if ICC was used to validate the robustness of phenotypes, the subjectivity of manual segmentation remained unavoidable, which may require a comparison between semi- and fully automated segmentation methods.

## Conclusions

In conclusion, DLRN on DCE-MRI images provided a non-invasive and practical method to preoperatively predict breast cancer LNM, thus potentially identifying appropriate axillary treatment options for patients with early breast cancer. In addition, the DLRN MRI phenotypes associated with LNM were associated with different biological pathways. Depending on the underlying biological pathways, MRI phenotypes may reflect the profile of key biological processes related to tumor proliferation, immune response, cellular communication, and signal transduction. These genetic and molecular acquisition functions contribute to improved clinical outcomes of patients. The future construction of prospective large-sample DLRN models will be essential to confirm this finding.

## Data Availability

The data sets generated and/or analyzed by the current study can be obtained from the corresponding author upon reasonable request. Source of public database data: TCGA, https://portal.gdc.cancer.gov/; TCIA, https://www.cancerimagingarchive.net/.
